# Tobacco Use and Suicide Attempt: Longitudinal Analysis with Retrospective Reports

**DOI:** 10.1371/journal.pone.0122607

**Published:** 2015-04-07

**Authors:** Ivan Berlin, Jahn K. Hakes, Mei-Chen Hu, Lirio S. Covey

**Affiliations:** 1 Département de Pharmacologie, Hôpital Pitié-Salpêtrière-Assistance publique-Hôpitaux de Paris-Faculté de médicine, Université P.& M. Curie—INSERM U1178, Paris, France; 2 Center for Administrative Records Research and Applications, U.S. Census Bureau, Suitland, MD, United States of America; 3 New York State Psychiatric Institute, Department of Psychiatry, Columbia University Medical Center, New York, NY, United States of America; Centre for Addiction and Mental Health, CANADA

## Abstract

**Background:**

Suicide has been associated with smoking/tobacco use but its association of and change in smoking/tobacco use status with suicide attempt (SA) is not well established.

**Methods:**

We investigated whether persistent, former tobacco use, initiation, quitting tobacco use, relapse to tobacco use, and DSM-IV nicotine dependence predict independently SA using Wave 1 and 2 data of the National Epidemiologic Survey of Alcohol and Related Conditions. Data from 34,653 US adults interviewed at Wave 1 (2001-02) and Wave 2 (2004-05) were analyzed. The main outcome measure was SA between Wave 1 and Wave 2 as reported at Wave 2.

**Results:**

Among the 1,673 respondents reporting lifetime SA at Wave 2, 328 individuals reported SA between Wave 1 and Wave 2. Current and former tobacco use at Wave 1 predicted Wave 2 SA independently of socio-demographic characteristics, psychiatric history, and prior SA (Adjusted Odds Ratio (AOR): 1.49; 95% CI: 1.13-1.95, AOR: 1.31; 95% CI:1.01-1.69, respectively versus never tobacco users). The strongest association with SA was observed among former tobacco users who relapsed after Wave 1 (AOR: 4.66; 95% CI: 3.49-6.24) and among tobacco use initiators after Wave 1 (AOR: 3.16; 95% CI: 2.23-4.49). Persistent tobacco use (current tobacco use at both Wave 1 and Wave 2) also had an increased risk of SA (AOR: 1.89; 95% CI: 1.47-2.42). However, former tobacco users in both Waves 1 and 2 did not show a significantly elevated risk for SA in Wave 2 (AOR:1.09, 95% CI: 0.78-1.52) suggesting that the risk resided mainly in Wave 1 former tobacco users who relapsed to tobacco use by Wave 2. DSM-IV nicotine dependence did not predict SA at Wave 2.

**Conclusion:**

In a representative sample of US adults, relapse, tobacco use initiation, and persistent tobacco use, which are amenable to intervention, were associated with risk of SA.

## Introduction

Suicide is a preventable public health problem with a million deaths per year worldwide. It is one of the leading causes of death among persons aged 15–44 years [1,2]. Knowledge and understanding of suicide risk factors may help to develop strategies for reducing the risk of this self-destructive behavior.

Roughly one billion persons worldwide are smokers [3]. Smoking reduces life expectancy by 10 to 20 years [4,5] mainly from cardiovascular, pulmonary diseases and cancer. Smoking prevalence is higher among men than among women and is highest among young men (<35 year) [6]. An independent association between smoking and completed suicide has been established in prospective cohort studies [7,8] however, an independent association between smoking and non-fatal suicide related outcomes (SROs: suicidal ideation, suicidal plan, and suicide attempt) remains unclear. Of eight previous longitudinal studies that controlled for potential demographic and psychiatric confounders, four found that current smoking was independently associated with SROs [9–12], four studies did not [13–16].

Suicide attempt is of specific interest. It is the behavior most proximal to suicide and is a major risk factor for completed suicide (along with mental or physical illness, physical or psychological pain, isolation, losses (relationship, work, financial)) [17]. Furthermore, suicide attempt itself often causes significant social disturbances to the person’s milieu including family, police and hospital services. Suicide attempts have been associated with nicotine dependence [13,18–20], current smoking [19,20], heavy smoking (20+ cigarettes smoked daily) [15], and with longer duration of smoking (14 years or more) [21]. In some [13,15,19] but not in all studies [18,21] adjustment for substance use, Axis I and Axis II mental disorders nullified this relationship. Tobacco use behavior status can change over time (e.g., current to former tobacco use, former to current tobacco use). To our knowledge, the risk of a suicide attempt in association with such changes has not been previously reported.

The aim of the present study was to investigate the association of tobacco use status (current or past tobacco use) and changes in tobacco use status occurring between the Wave 1 and the Wave 2 interviews with suicide attempt while controlling for potential confounding effects of socio-demographic and psychiatric risk factors utilizing data from a two-wave psychiatric epidemiological survey of a representative sample of U.S. adults. We also compared the strength of the association of tobacco use status and changes in tobacco use status with nicotine dependence in predicting suicide attempt. Nicotine dependence as defined by DSM-IV criteria is based on several behavioral components [22]; the concepts of tobacco use status and change in tobacco use status are defined more narrowly to actual tobacco use or non-use. Comparison of DSM-IV nicotine dependence versus definitions reflecting tobacco use may allow a determination of whether the potential association is related specifically to tobacco use or not.

## Materials and Methods

### Ethics statement

The US Census Bureau and the US Office of Budget and Management approved the NESARC protocol. More comprehensive accounts of the NESARC procedures are available elsewhere [25]. As per US Census Bureau regulations, all participants gave written informed consent; all NESARC data are classified as restricted access and are available only to persons of Special Sworn Status who are trained to maintain confidentiality of personally-identifiable and other sensitive information. The data are only accessed after administrative approval of a formal analysis request, and all output are inspected to verify that there is no disclosure of sensitive information. Participants’ records/information was anonymized and de-identified prior to analysis.

The National Epidemiologic Survey of Alcohol and Related Conditions (NESARC) interviews were conducted with persons in households, military personnel living off base, and individuals living in group quarters such as boarding houses, shelters, and college dorms. The aim of NESARC was to assess alcohol and other substance use and mental disorders according to Diagnostic and Statistical Manual of Mental Disorders, 4th. Edition (DSM-IV) [22]. Data were collected to obtain a representative national sample of US adults. In Wave 1 (2001–2002), face-to-face interviews were completed with 43,093 persons aged 18 years or older. Blacks, Hispanics, and young adults (18–24 years) were oversampled. The Wave 1 sample was re-interviewed in Wave 2 (2004–2005) three years later (mean interval = 36.6 months, s.d. = 2.62) [23] with an overall response rate based on the Wave 1 sample of 86.7% (N = 34,653). Details of the NESARC Wave 1 and Wave 2 studies have been described extensively elsewhere [9,24,25].

For both Waves 1 and 2, the AUDADIS-IV (Alcohol Use Disorder and Associated Disabilities Interview Schedule-IV) was administered by interviewers from the US Census Bureau. The reliability and validity of the DSM-IV diagnoses and nicotine dependence [26] obtained through the AUDADIS-IV have been demonstrated in clinical and general samples in the USA and in other countries [27]. The kappa value of nicotine dependence assessed by AUDADIS-IV is 0.60 [24]. Although Attention-Deficit/Hyperactivity Disorder (ADHD) was included only in the Wave 2 interview, we used it as a predictor because a) DSM-IV [22] states that its essential feature is its persistent pattern and it was unlikely that, among adults, diagnosed at Wave 2, it was not present at Wave 1 with a mean interval between Wave 1 and Wave 2 being 3.6 years; and b) it is a known risk factor for both smoking and suicide and its omission could have been a higher level of error than its inclusion among the predictors.

Interview data for this study were obtained from all Wave 2 respondents (N = 34,653). All Wave 2 participants were asked the question: “In your entire life did you ever attempt suicide?” This question yielded 1,265 positive responses as mentioned in a previous cross sectional analysis [18]. Persons reporting low mood were further queried during both Waves 1 and 2 regarding the occurrence of suicide related outcome such as suicide ideation, wish to die and suicide attempt. The questions for low mood at the Wave 2 interview, were: “Since your LAST interview in (month/year), have you ever had a time when you felt sad, blue, depressed, or down most of the time for at least 2 weeks?” and “Since your LAST interview, have you ever had a time, lasting at least 2 weeks, when you didn’t care about the things that you usually cared about, or when you didn’t enjoy the things you usually enjoyed?” Responses to these questions identified 7352 individuals considered as having low mood [9]. Several of these participants (N = 408) reported suicide attempt only in the section on “low mood”, but did not answer positively to the specific lifetime suicide attempt question asked of all study subjects in Wave 2. These additional reports of suicide attempt at Wave 2 were added to the number of positive responses to the specific lifetime suicide attempt question, resulting in a total of 1673 Wave 2 respondents with a lifetime suicide attempt. Among the 1673 Wave 2 respondents reporting lifetime suicide attempt, 328 individuals reported at least one suicide attempt that occurred between Wave 1 and Wave 2; among them 213 individuals reported having a previous suicide attempt before Wave 1, 115 individuals had made suicide attempt(s) only between Wave 1 and 2. Between-waves occurrence of suicide attempt(s) was ascertained by comparing the respondent’s age at most recent suicide attempt with age at Wave 1.

Tobacco use categories were based on responses to the Wave 1 question regarding lifetime tobacco use of at least 100 cigarettes, at least 50 cigars, smoked a pipe at least 50 times, used snuff at least 20 times or used chewing tobacco at least 20 times. Those who never used tobacco had responded “No” to all of these questions; tobacco users had responded “Yes” to one or more of these questions; former tobacco users had used tobacco but not in the past 12 months; and current tobacco users had smoked or used tobacco within the past 12 months. At the Wave 2 interview, these same questions on tobacco use were asked with regard to the period since the last interview (in month/year). Nicotine dependence was defined following the DSM-IV criteria [22]. Both the NESARC instrument and the frequency statistics in Table 1 (see below) use the respondent as the observational unit.

**Table 1:** Weighted Percentages, Standard Errors, Unadjusted and Adjusted Odds Ratios (OR and AOR) and 95% Confidence Intervals (CI) of Suicide Attempts (SA) Occurred Between Wave 1 and 2 (mean: 3.1 years) by Wave 1 Characteristics.

Variable	SA (n)	Total sample of each charactteristic(N)	Mean weighted SA rate	Std Error	OR	95% CI	AOR^§^	95% CI
**Race/ethnicity**								
White (non-Hispanic)	163	20,174	0.81	0.05	1.00		1.00	
Black	52	6,432	0.77	0.08	0.95	(0.75, 1.20)	0.85	(0.70, 1.04)
Asian/Pacific Islander	11	1,000	0.69	0.12	0.86	(0.60, 1.23)	1.32	(0.83, 2.11)
American Indian/Alaskan Native	16	691	1.88	0.42	2.36	(1.47, 3.79)	1.43	(0.75, 2.71)
Hispanic	86	6,356	1.34	0.03	1.67	(1.48, 1.88)	2.02**	(1.76, 2.32)
**Gender** (reference: male)								
Female	216	20,089	1.06	0.04	1.53	(1.31, 1.78)	1.16	(0.98, 1.36)
**Age** (years)								
18 to 19	26	1,143	2.09	0.31	1.00		1.00	
20 to 29	79	5,576	1.33	0.12	0.63	(0.46, 0.88)	0.65*	(0.43, 0.98)
30 to 44	141	11,013	1.25	0.06	0.60	(0.44, 0.81)	0.73	(0.47, 1.13)
45 to 64	71	10,917	0.50	0.04	0.24	(0.17, 0.33)	0.37**	(0.23, 0.61)
65 and over	11	6,004	0.13	0.03	0.06	(0.03, 0.10)	0.24**	(0.12, 0.48)
**Marital Status**								
Married	113	17,401	0.65	0.04	1.00		1.00	
Cohabiting	12	1,012	1.01	0.24	1.56	(0.95, 2.55)	0.57*	(0.36, 0.90)
Widowed	11	3,056	0.28	0.04	0.42	(0.31, 0.58)	0.50**	(0.33, 0.75)
Divorced	53	4,369	1.18	0.09	1.81	(1.54, 2.13)	0.67**	(0.52, 0.86)
Separated	34	1,139	3.12	0.45	4.90	(3.65, 6.58)	1.21	(0.81, 1.79)
Never married	105	7,676	1.38	0.09	2.14	(1.80, 2.54)	0.98	(0.77, 1.26)
**Household Income**								
Below $20,000	138	8,959	1.53	0.11	1.00		1.00	
$20,000 to $35,000	86	7,309	1.18	0.08	0.77	(0.63, 0.93)	0.98	(0.76, 1.26)
$35,000 to $60,000	51	8,812	0.62	0.06	0.40	(0.31, 0.51)	0.61**	(0.44, 0.85)
Over $60,000	53	9,573	0.53	0.03	0.34	(0.28, 0.41)	0.65**	(0.51, 0.83)
**Employment**								
Not unemployed	245	31,806	0.74	0.03	1.00		1.00	
Unemployed	83	2,847	2.74	0.20	3.77	(3.20, 4.43)	1.31**	(1.07, 1.62)
**Region**								
Northeast	64	6,444	0.75	0.03	1.00		1.00	
Midwest	64	7,540	0.84	0.09	1.12	(0.90, 1.39)	1.07	(0.85, 1.35)
South	123	12,833	1.03	0.08	1.38	(1.16, 1.64)	1.34**	(1.12, 1.61)
West	77	7,836	0.82	0.03	1.09	(0.99, 1.21)	1.17*	(1.00, 1.36)
**Urbanicity** (reference: rural)								
Urban	140	11,672	1.10	0.03	1.38	(1.22, 1.57)	1.32**	(1.17, 1.48)
**Educational attainment**								
No high school diploma	86	5,744	1.23	0.12	1.37	(1.10, 1.71)	1.03	(0.84, 1.28)
High school diploma	99	9,955	0.90	0.06	1.00		1.00	
Some college or more	143	18,954	0.79	0.04	0.87	(0.75, 1.02)	1.26*	(1.03, 1.53)
**Lifetime psychiatric disorder**								
**Axis I disorders**								
Alcohol Use Disorder	151	9,937	1.36	0.09	2.03	(1.74, 2.36)	1.06	(0.86, 1.29)
Substance Use Disorder	94	3,420	2.51	0.21	3.68	(3.07, 4.41)	0.93	(0.75, 1.15)
Anxiety disorders	136	6,327	2.25	0.13	3.90	(3.36, 4.52)	1.30**	(1.08, 1.57)
Mood disorders	197	7,621	2.46	0.13	5.42	(4.80, 6.13)	0.89	(0.71, 1.11)
Attention deficit-hyperactivity disorder (ADHD)	54	807	5.43	0.63	7.42	(5.78, 9.52)	1.02	(0.70, 1.48)
**Axis II Disorders**								
Borderline	195	2,231	9.12	0.40	27.1	(24.0, 30.6)	7.54**	(6.08, 9.34)
Schizotypal	108	1,534	7.00	0.56	11.8	(9.79, 14.3)	1.28	(0.99, 1.66)
Narcissistic	77	2,449	2.50	0.17	3.27	(2.82, 3.79)	0.52**	(0.42, 0.64)
Avoidant	62	821	7.72	0.78	11.5	(9.27, 14.3)	1.45**	(1.13, 1.87)
Antisocial	52	1,154	3.72	0.49	4.93	(3.69, 6.58)	1.07	(0.76, 1.50)
Dependent	21	147	17.6	3.38	26.0	(16.6, 41.0)	1.95**	(1.22, 3.11)
Obsessive/Compulsive	64	2,753	2.06	0.24	2.68	(2.11, 3.41)	0.66**	(0.52, 0.85)
Paranoid	85	1,689	5.00	0.40	7.49	(6.31, 8.89)	1.10	(0.88, 1.38)
Schizoid	50	1,144	5.06	0.57	7.03	(5.58, 8.84)	1.30	(1.04, 1.63)
Histrionic	30	651	4.76	0.76	6.10	(4.37, 8.51)	0.83	(0.60, 1.16)
**Previous (before Wave 1) suicide attempt**	213	1,558	13.4	0.65	44.8		13.99**	(11.8, 16.6)
**Smoking status** ^#^								
Current tobacco user	167	8,900	1.69	0.10	2.90	(2.41, 3.49)	1.49**	(1.13, 1.95)
Former tobacco user	42	6,641	0.57	0.05	0.96	(0.78, 1.18)	1.31*	(1.01, 1.69)
Never used tobacco	119	19,112	0.59	0.04	1.00		1.00	

(N = 34,653).

^#^OR estimates from logistic regression of Wave 2 suicide attempts on Wave 1 current smoker or former smoker, and all categories of control variables except smoking status changes.

^§^OR estimates from multiple logistic regression of Wave 2 suicide attempt on smoking status changes, race and ethnicity, sex, and Wave 1 measurements for age group, marital status, household income group, unemployment, census region, urbanicity, educational group, previous suicide attempt(s) and psychopathology.

* significant at alpha ≤0.05

** significant at alpha ≤0.01

A change in tobacco use status variable from Wave 1 to Wave 2 was created with the following categories according to the reports of smoking at Waves 1 and 2: (1) never tobacco user to never tobacco user (i.e. never tobacco user), (2) former tobacco user to former tobacco user (i.e. long-term former tobacco user), (3) current tobacco user to former tobacco user (recent former tobacco user, i.e. quitter), (4) current tobacco user to current tobacco user (i.e. persistent tobacco user), (5) never tobacco user to current tobacco user (i.e. tobacco use initiator or new tobacco user), and (6) former tobacco user to current tobacco user (i.e. relapser).

### Data analysis

Weighted percentages and survey-adjusted standard errors measured the distribution of the covariates (demographic characteristics and lifetime psychiatric variables) reported at Wave 1 for the sample that completed the Wave 2 interview. Univariate and multivariate logistic regressions with survey-design corrections and sampling weights were used to estimate the likelihood of suicide attempts occurring between Wave 1 and Wave 2. The resulting estimates are presented as unadjusted (OR) and adjusted odds ratios (AOR), respectively, with 95% confidence intervals (CI). All multivariate analyses included all demographic and lifetime psychiatric diagnostic variables (control variables) and a previous suicide attempt (before Wave 1), as shown in Table 1. The comparative strength of DSM-IV nicotine dependence versus tobacco use status (current, past, or never) and change in smoking status was tested by the Bayesian information criterion (BIC), the covariances between estimated parameters, and likelihood ratio (LR) tests on the improvements in the -2 Log-Likelihood among two nested models [28]. Data were analyzed with SAS 9.2 (Cary, NC, USA) using the procedures included to correct for complex survey design.

## Results

### Prevalence of suicide attempt between Waves 1 and 2

The 328 respondents reporting suicide attempts for the period between Wave 1 and Wave 2 interviews correspond to a weighted proportion of 0.88% (s.e. = 0.04%) of the Wave 2 sample of 34,653 persons. Table 1 shows the number of suicide attempts, the weighted suicide attempt rates by Wave 1 smoking status, demographics, and lifetime psychiatric disorders, and the unadjusted and adjusted odds ratios indicating risk for a future suicide attempt.

### Predictors of suicide attempt between Waves 1 and 2

After controlling for other factors, the strongest predictor of suicide attempt between Wave 1 and Wave 2 was previous suicide attempt (Table 1). The risk of suicide attempt was significantly higher at the 95% confidence level among Hispanics and college educated persons (Table 1). Unemployment as of the Wave 1 interview, and urban residency (*versus* rural) were also associated with increased suicide attempts. The risk of a suicide attempt decreased with increasing age and increasing household income. Of the Axis II diagnoses, borderline, avoidant, and dependent personality disorders were positive predictors of significantly increased suicide attempt risk, whereas narcissistic and obsessive-compulsive personality disorders were associated with significantly lower suicide attempt risk. Among Axis I disorders, only anxiety disorders were associated with a significantly increased adjusted risk of suicide attempt. In the unadjusted analyses, alcohol use disorder, substance use disorder, mood disorders, and ADHD (and also female gender) were significantly related to suicide attempts, but not in the adjusted analyses. This can be attributed to the significant bivariate correlations with previous (r = 0.61) and recent (r = 0.373) suicide attempts and with other study covariates in polychloric correlational analyses (data not shown).

Adjusted for all potential confounders, compared to never tobacco use, current tobacco use and former tobacco use status at Wave 1 were significantly associated with suicide attempts occurring between Wave 1 and Wave 2. Compared to never tobacco use, current tobacco users had an estimated 49% higher risk of attempting suicide; a 31% increased risk of suicide attempt was also observed among former tobacco users (bottom of Table 1.) (Confidence intervals for all point estimates are presented in the Tables. The point estimates described in the text are all statistically significant unless otherwise noted.)

Table 2 shows suicide attempt risk associated with changes in tobacco use status between Wave 1 and 2. Compared to never tobacco users by Wave 2, persistent tobacco users had an estimated 89% higher risk of attempting suicide. The association is stronger for new tobacco users (started using tobacco between Wave 1 and Wave 2), a more than 2-fold increased risk. The strength of this association was greater than the association among persistent tobacco users (χ^2^ test versus persistent tobacco users: 5.72, p = 0.02).

**Table 2:** Weighted Percentages, Standard Errors, Unadjusted and Adjusted Odds Ratios (OR) and 95% Confidence Intervals of Suicide Attempts (SA) Occurred Between Wave 1 and 2 (mean: 3.1 years) according to changes in smoking status during the same period.

Smoking status in Wave 1 to Wave 2	SA (n)	Total sample (N)	Mean weighted SA rate	Std Error	Unadjusted OR	95% confidence intervals	Adjusted OR	95% confidence intervals
Never used tobacco (in Wave 1 and 2)	103	18,356	0.52	0.04	1.00		1.00	
Long-term former tobacco user (in Wave 1 and 2)	30	6,303	0.41	0.05	0.80	(0.60, 1.07)	1.09	(0.78, 1.52)
Recent former tobacco user (current tobacco user in Wave 1, former tobacco user in Wave 2)	17	2,031	0.60	0.12	1.16	(0.71, 1.89)	0.88	(0.46, 1.70)
Persistent tobacco user (in Wave 1 and 2)	150	6,869	2.01	0.12	3.95	(3.28, 4.76)	1.89**	(1.47, 2.42)
New tobacco user (never used tobacco in Wave 1, current tobacco user in Wave 2)	16	726	2.43	0.46	4.80	(3.20, 7.20)	3.16**	(2.23, 4.49)
Relapser (former tobacco user in Wave 1, current tobacco user in wave 2)	12	338	3.23	0.21	6.43	(5.35, 7.73)	4.66**	(3.49, 6.24)

(N = 34,653).

Adjusted for all variables listed in Table 1 (except for the three “Smoking status” variables).

** significant at alpha ≤0.01

A breakdown of Wave 1 former tobacco users, however, suggests that the risk for suicide attempt resides mainly in Wave 1 former tobacco users who relapsed to tobacco use in Wave 2 (AOR:4.66, 95% CI: 3.49, 6.24); the long-term former tobacco users (former tobacco users both at Wave 1 and Wave 2) did not show a significantly elevated risk for suicide attempt in Wave 2 (AOR:1.09, 95% CI: 0.78, 1.52).

### Comparison of tobacco use status, tobacco use status change, and nicotine dependence as predictor of suicide attempt

Four regression models were run to assess the strengths of associations of DSM-IV nicotine dependence, tobacco use status or tobacco use status change with suicide attempt, respectively (Table 3).

**Table 3:** Comparison of lifetime nicotine dependence and different tobacco use variables in the multiple logistic regression models predicting Wave 1 to Wave 2 suicide attempt.

Predictors from multiple logistic regression model	-2 log L	Adjusted odds ratios (95% confidence intervals) for suicide attempt
Model 1. DSM-IV nicotine dependence (ND)*	13,631, 353	ND: 1.15 (0.93,1.40) p = 0.19
**Model 2. Tobacco use status**	13,597, 652	
Current tobacco user (CTU) at Wave 1		CTU: 1.49 (1.13,1.94) p = 0.004
Former tobacco user (FTU) at Wave 1		FTU: 1.31 (1.01, 1.69) p = 0.04
**Model 3A. Tobacco use status and DSM IV nicotine dependence**	13,594,531	ND: 0.87 (0.69, 1.11)
		p = 0.27
		CTU: 1.61 (1.17, 2.21) p = 0.004
		FTU: 1.35 (1.04, 1.76) p = 0.025
**Model 3B. Change in tobacco use status between Wave 1 and 2 and DSM IV nicotine dependence**	13,418,650	ND^#^: 0.81 (0.64, 1.03) p = 0.09

Adjusted for all covariates listed in Table 1.

*Reference group: No nicotine dependence, controlling for all other variables.

^#^ For Model 3B, the five individual coefficients for tobacco use status change (from Wave 1 to Wave 2, see bottom of Table 1) are not presented; AOR values range from 0.97 to 4.98, and have the same pattern of statistical significance for the AORs as shown in Table 1.

DSM-IV nicotine dependence was not associated with suicide attempt (Model 1). Current and former tobacco use in Wave 1were associated with the occurrence of suicide attempt between the Wave 1 and Wave 2 interviews (Model 2). When DSM-IV nicotine dependence and tobacco use status were included in the same model (Model 3A), both current tobacco use and former tobacco use but not nicotine dependence were associated with suicide attempt risk. Finally, when DSM-IV nicotine dependence and tobacco use status changes between Wave 1 and 2 were included in the same model (Model 3B), tobacco use status changes but not DSM-IV nicotine dependence was associated with suicide attempt risk. Thus, DSM-IV nicotine dependence alone or in combination with either tobacco use status or changes in tobacco use status did not improve the models predicting suicide attempt.

## Discussion

This longitudinal analysis using responses obtained retrospectively during the research interviews found that current and past tobacco use are significant independent predictors over a 3-year period of a future suicide attempt. This finding is in line with two previous reports [18,21], but contradicts two other studies [13,19]. Tobacco use status changes exerted predictive effects on suicide attempt. Relapse to tobacco use and initiation of tobacco use were strongly associated with suicide attempt. However, former tobacco use status both at Wave 1 and Wave 2 was not associated with increased suicide attempt risk. DSM-IV nicotine dependence did not predict a suicide attempt during Wave 2.

Traditional risk factors for suicide attempt are alcohol and drug abuse, depression, schizophrenia, unemployment, sociopathy, hostility and living alone [17]. A cross sectional WHO survey involving 21 countries and using similar DSM-IV criteria as the current report identified anxiety, mood, impulse conduct disorders, including ADHD, and substance use disorders such as alcohol use and dependency, drug abuse and dependency, but not tobacco use, as associated with suicide attempt [29]. Cross sectional analysis of the Wave 2 of the NESARC data found depressive disorder, borderline personality disorders, post-traumatic stress disorder, and nicotine dependence as the mental disorders most strongly associated with suicide attempt [18]. The prospective study from Finland identified the same demographic predictors as the current analysis: younger age, female gender, low socioeconomic level, and living alone, and also that suicide attempt risk was higher among both current smokers and ex-smokers than among non-smokers both in men and women [21].

### Tobacco smoking, smoking abstinence and suicide attempt

Current tobacco use, occurring among relapsers persistent, d, or new tobacco users, was independently associated with increased risk of suicide attempt, supporting a previous prospective study[21] and cross-sectional findings in depressed individuals [30]. The findings regarding tobacco abstinence reveal a nuanced effect. Long-term former tobacco users (former tobacco users in Wave 1 and Wave 2) did not show a significantly elevated relative risk for suicide attempt in Wave 2 but former tobacco users at Wave 1 who relapsed by Wave 2 did. An effect of relapse and new smoking on SROs had been seen in a previous analysis by our group in the NESARC data restricted to the low mood sample: Wave 1 former smokers who relapsed to smoking in Wave 2 showed the highest risk for SROs at Wave 2; significant risk for a future SRO was seen in Wave 1 never smokers who began smoking during Wave 2 [9]. The current analysis extends those prior findings to the general population, not only persons with low mood.

### Nicotine dependence and suicide attempt

The current study did not find a significant association between DSM-IV nicotine dependence and suicide attempt. This finding corresponds with data from 5,001 respondents in the National Comorbidity Survey (1990–1992) [13] and its follow up survey (2001–2003) [19] but differs from two cross-sectional studies, both using the NESARC, which found that nicotine dependence was associated with suicide attempt in the Wave 1 [20] and in the Wave 2 sample [18]. Differences in the longitudinal design of the present study based on NESARC data and the National Comorbidity Survey [13,19] versus the cross-sectional analyses [18,20] may account for the contradictory results. Moreover, nicotine dependence as defined by DSM-IV and tobacco use are two alternative approaches to the issue of the association of tobacco use with suicide attempt. The first one is based on behavioral patterns, the second one on the pharmacological effects of substance use. The two obviously may have an overlap, but our data, that uniquely allow such a comparison, show that tobacco use/consumption is more predictive of suicide attempt than DSM-IV nicotine dependence.

### Potential pathophysiological mechanisms

The independent associations of persistent tobacco use, relapse to tobacco use, and initiation of tobacco use with suicide attempt raise neurobiologial and genetic hypotheses worth mentioning. A) Tobacco and nicotine are powerful modifiers of central neurotransmitter systems [31]. Smoking is associated with reduced MAOA and B activities leading to increased MAO-dependent neurotransmitter availability [32]. Genetic variations of MAOA are associated with impulse control [33]. Low expression of the MAOA gene variant is associated with differences in limbic circuitry for emotion regulation and cognitive control that may be involved in the association of MAOA with impulsive aggression [34]. Both smoking induced reduction in MAOA activity or low MAOA expression may increase impulsive behavior. As shown in prior research among patients with psychiatric disorders, smokers are more likely to make a suicide attempt and cigarette consumption had an inverse dose-dependent relationship with serotonin function [35]. Among smokers with major depression or bipolar depression, lower CSF MHPG (3-methoxy-4-hydroxyphenylglycol, a MAO dependent norepinephrine metabolite) has been found to predict short-term risk of suicide attempt [36]. B) Suicide rates are elevated among those living in high altitudes [37,38], and it has very recently been reported that chronic obstructive pulmonary disease, a chronic hypoxic state, is associated with suicide attempt in a Korean population [39]. The possibility has recently been raised [40,41] that mild hypoxia, or inflammatory processes [42] both inevitably associated with smoking/tobacco use, may be one of the underlying mechanisms of the smoking/tobacco use—SROs /suicide relationship. These possible pathophysiological mechanisms are, as of today, certainly speculative but offer new research directions for understanding the dynamics of tobacco use, nicotine dependence, and their relationship with suicidal behaviors. Related neurobiological and genetic underpinnings also warrant further investigations.

### Strengths and limitations

#### Limitations

The sample does not include individuals younger than 18 years old, a population particularly at risk of both tobacco use initiation and suicidal behavior. Because other SROs such as suicide ideation or wish to die were not questioned from all Wave 2 participants, comparisons among suicide attempt and other SRO could not be performed. The lack of the exact date of the suicide attempts and tobacco use status changes does not allow a prospective analysis, precluding inferences regarding a causal relationship. This study did not assess the reverse predictive relationships of a prior suicide attempt with the tobacco use variables. The possibility cannot be discounted that a suicide attempt or a concurrently occurring psychiatric disorder provoked a return to tobacco use or resorting to tobacco use by a never tobacco user. Because of the low cell counts for smoking change variables, interactions could not be tested but may exist. This study used only Wave 1 demographic and psychiatric covariate predictors; changes in those characteristics may be linked to changes in tobacco use status and suicide attempt. Because life stressors can impact both tobacco use and suicide attempt, further studies should include data about psychosocial stressors. Tobacco use status assessment was based on self-report and was not biochemically validated. Although the data analysis plan foresaw to look at the suicide attempt risk according to the amount of tobacco used through the question “usual quantity smoked per day”, because of the low cell counts by tobacco use per day and the consequent loss of power, data were only analyzed by tobacco use status. The number of non-cigarette-smoking tobacco users was very low (4/328 suicide attempters), so data could not be analyzed to assess the difference between cigarette smokers and those using other forms of tobacco in association with suicide attempt. Finally, the confounding role of psychotropic medications could not be assessed as information on drug use had not been systematically obtained.

#### Strengths

Strengths of this analysis include the large sample size, the longitudinal design, and the rigorous face-to-face interviews based on a well-validated diagnostic instrument (AUDADIS-IV) [26,27]. A further strength is that all Wave 2 participants were asked a question about suicide attempt, even those who did not report previous low mood. The use of a representative national sample allowed some generalization to the US population as a whole, the NESARC having been specifically sampled and weighted to represent it. The suicide attempt incidence rate in this study is of 8.8/1000. Data from the National Hospital Ambulatory Medical Care Survey, for attempted suicide or self-inflicted injury during 1997 to 2001 showed an annual visit rate of 1.5 (95% CI: 1.3, 1.7) visits per 1,000 US citizens [43] which corresponds to a rate of 4.6/1000 for a period of 3.6 years covered by the current study. Although this rate is somewhat lower than the suicide attempt rate observed in the present study, it is of the same order and provides an external validity.

## Future Impact of the Findings and Conclusions

Past suicide attempt is the strongest predictor of a future suicide attempt [17,44–47]. If further studies confirm that tobacco use status changes are independent predictors of suicide attempt, pro-active interventions to prevent relapse and tobacco use initiation may reduce suicide attempt risk. A true prospective design that enables a clear indication of the relative timing between tobacco use status change and suicide attempt is needed to clarify the role of cessation of tobacco use as a preventive tactic for reducing the risk of future suicide attempt. Even small increases in the rate of suicide attempt caused by tobacco use may have a major public health importance and as has recently been demonstrated, tobacco control interventions may effectively reduce suicide risk [48].
